# Supplementary material for: Membrane to cortex attachment determines different mechanical phenotypes in LGR5+ and LGR5- colorectal cancer cells
Source: Nat Commun. 2024 Apr 18;15:3363. doi: 10.1038/s41467-024-47227-2 (PMC11026456; doi:10.1038/s41467-024-47227-2)
Supplement: Supplementary file 1 — Supplementary Information [file 41467_2024_47227_MOESM1_ESM.pdf]

Supplementary Material

Membrane to cortex attachment determines different mechanical phenotypes in LGR5+ and LGR5- colorectal cancer cells

Sefora Conti[1], Valeria Venturini[2], Adrià Cañellas-Socias[3, 4], Carme Cortina[3, 4], Juan F. Abenza[1], Camille Stephan-Otto Attolini[3], Emily Middendorp Guerra[3, 4], Catherine K Xu[5], Jia Hui Li[6], Leone Rossetti[1], Giorgio Stassi[7], Pere Roca-Cusachs[1,8], Alba Diz-Muñoz[6], Verena Ruprecht[2,9,10], Jochen Guck[5,11,12], Eduard Batlle[3, 4,10]*, Anna Labernadie[1,13]*, Xavier Trepat[1,8,10,14]*

1. Institute for Bioengineering of Catalonia (IBEC), The Barcelona Institute for Science and Technology (BIST), Barcelona, Spain.
2. Centre for Genomic Regulation (CRG), The Barcelona Institute for Science and Technology (BIST), Barcelona, Spain.
3. Institute for Research in Biomedicine (IRB Barcelona), Barcelona Institute of Science and Technology (BIST), Barcelona, Spain.
4. Centro de Investigación Biomedica en Red de Cancer (CIBERONC), Barcelona, Spain.
5. Max Planck Institute for the Science of Light, Erlangen, Germany.
6. Cell Biology and Biophysics Unit, European Molecular Biology Laboratory (EMBL), Heidelberg, Germany.
7. Department of Surgical Oncological and Stomatological Sciences, University of Palermo, Palermo, Italy
8. Facultat de Medicina, University of Barcelona (UB), Barcelona, Spain.
9. Universitat Pompeu Fabra (UPF), Barcelona, Spain.
10. Institució Catalana de Recerca i Estudis Avançats (ICREA), Barcelona, Spain.
11. Department of Physics, Friedrich-Alexander Universität Erlangen-Nürnberg (FAU), Germany
12. Max-Planck Zentrum für Physik und Medizin, Erlangen, Germany.
13. Centro de Investigación Principe Felipe (CIPF), Valencia, Spain.
14. Centro de Investigación Biomédica en Red en Bioingeniería, Biomateriales y Nanomedicina (CIBER-BBN), Barcelona, Spain.

Supplementary Figures

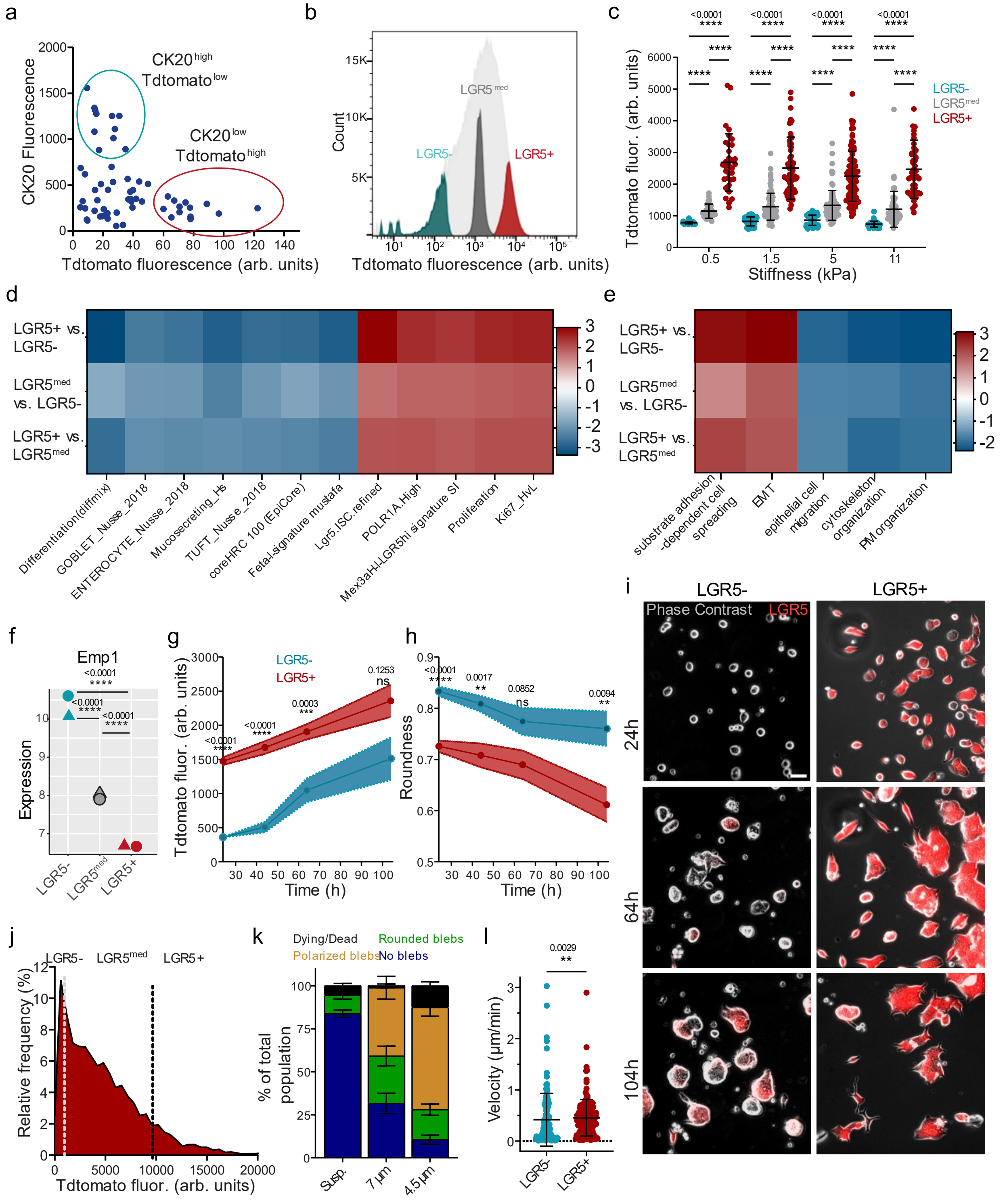

Supplementary Figure 1. Plasticity of PDO7 single cells, LGR5 Tdtomato fluorescence and response to confinement. a. Quantification of CK20 and Tdtomato fluorescence in CRC PDOs cultured for 1 week in culture matrix gel. **b.** Flow cytometric sorting strategy used to obtain LGR5-, LGR5^{med} and LGR5+ cells from CRC PDOs cultured for 1 week. **c.** Tdtomato fluorescence intensity of sorted cells 24 h after seeding on collagen I coated gels. Data are represented as the

mean ± s.d. of n > 73 cells/condition from four independent experiments. Statistical significance was determined using Shapiro-Wilk normality test, followed by a Kruskal-Wallis multiple-comparison test. (**d**-**e**). Heatmaps showing Normalized Enrichment Scores (NES) for selected gene sets (**d**) and GOBP gene sets (**e**) in LGR5-, LGR5^{med} and LGR5+ cells. Each row represents the comparative values of gene sets between two populations. NES and p-values were computed through a Roast-GSA analysis as described in the methods section. *p* values are listed in Supplementary Table 1. Gene lists of each gene signature are provided in Supplementary Data 1. For each sample biological duplicates were analysed. **f.** *Emp1* expression in sorted cells as quantified by RNA-seq. Statistical analysis was performed using t-tests. Duplicates for each population were analysed. (**g, h**) Change in Tdtomato fluorescence and roundness in sorted single cells as a function of time. n ≥ 86 cells from independent two experiments. Statistical significance was determined using two-way analysis of variance, followed by a Šidák multiple-comparison test. **i.** Time lapse of sorted single cells on 3kPa gels. Time 0 = 24 h after sorting. Representative images from two independent experiments. **j.** Tdtomato fluorescence intensity of LGR5-, LGR5^{med} and LGR5+ cells in RT-DC. **k.** Percentage of dying/dead, polarized blebs, rounded blebs and no blebs in PDO cells in suspension or under 7 and 4.5 µm confinement. Data are represented as the mean ± s.d. of n ≥ 30 positions/condition from one experiment. Statistical significance was determined using two-way analysis of variance, followed by a Šidák multiple-comparison test. **l**. Migration velocity of tracked nuclei of confined LGR5- and LGR5+ cells. Data are represented as the mean ± s.d. of n ≥ 110 cells/condition from four independent experiments. Statistical significance was determined using Shapiro-Wilk normality test, followed by a Kruskal-Wallis multiple-comparison test. Source data are provided as a Source Data file.

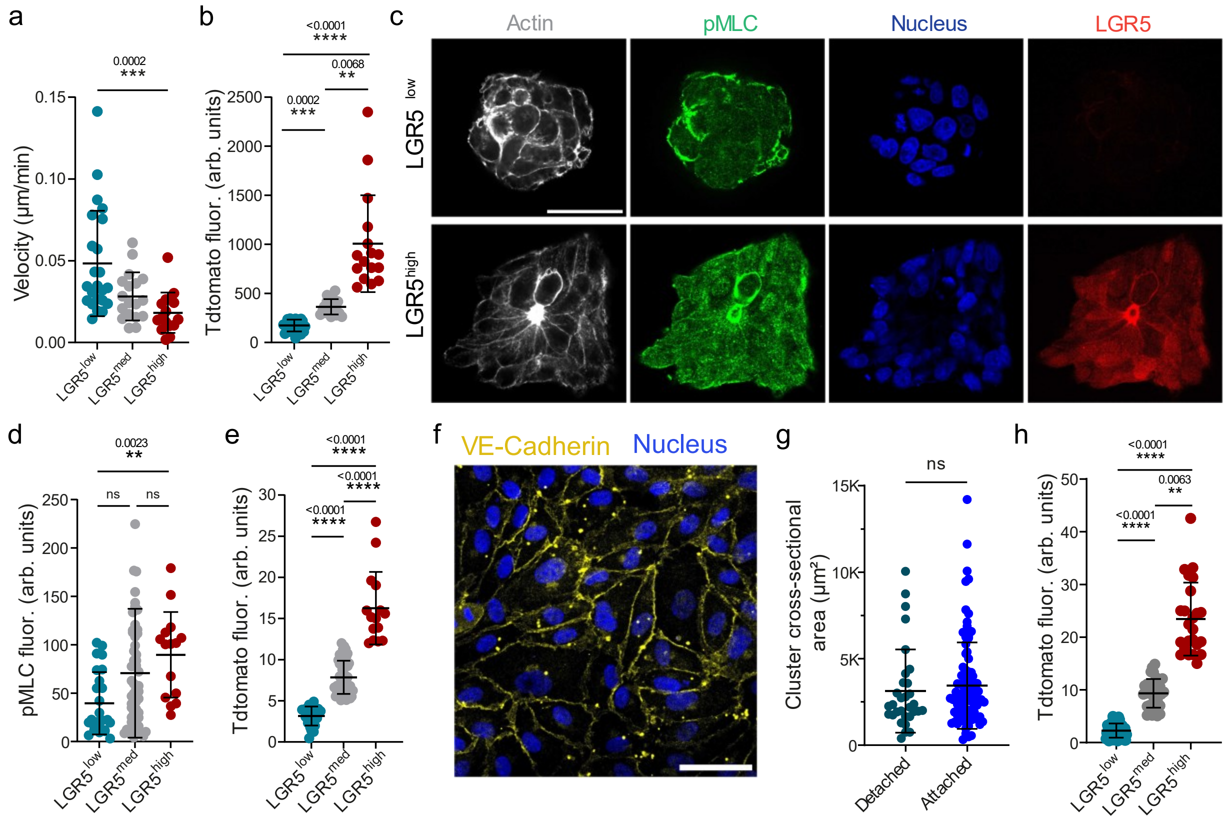

Supplementary Figure 2. Analysis of cluster dynamics. (a, b). Migration velocity (**a**) and Tdtomato fluorescence intensity (**b**) of clusters on 11kPa gels. Data are represented as the mean ± s.d. of n = 58 clusters from two independent experiments. Statistical significance was determined using Shapiro-Wilk normality test, followed by a Kruskal-Wallis multiple-comparison test. **c.** PDO clusters seeded on 3kPa gels coated with Collagen I. Clusters were stained for Actin, phosphorylated myosin light chain (pMLC) and nuclei (hoechst). LGR5+ cells are labelled with Tdtomato. Scale bar, 50 µm. (**d, e**) Quantification of mean pMLC (**d**) and Tdtomato fluorescence intensity (**e**) of $LGR5^{low}$, $LGR5^{med}$ and $LGR5^{high}$ clusters. Data are represented as the mean ± s.d. of n ≥ 15 clusters/subgroup from three independent experiments. Statistical significance was determined using Shapiro-Wilk normality test, followed by a Kruskal-Wallis multiple-comparison test. **f.** HUVEC monolayer grown for 4 days on collagen I coated gels and stained for VE-cadherin and nuclei (hoechst). Scale bar 50 µm. **g** Cross-sectional area of clusters that remained attached or detached from endothelial monolayer during the 15 h timelapse acquisition. **h.** Tdtomato fluorescence intensity of clusters divided into three groups. (**g, h**) Data are represented as the mean ± s.d. of n ≥ 24 clusters/subgroup from four independent experiments. Statistical significance was determined using Shapiro-Wilk normality test, followed by a Kruskal-Wallis multiple-comparison test. Source data are provided as a Source Data file.

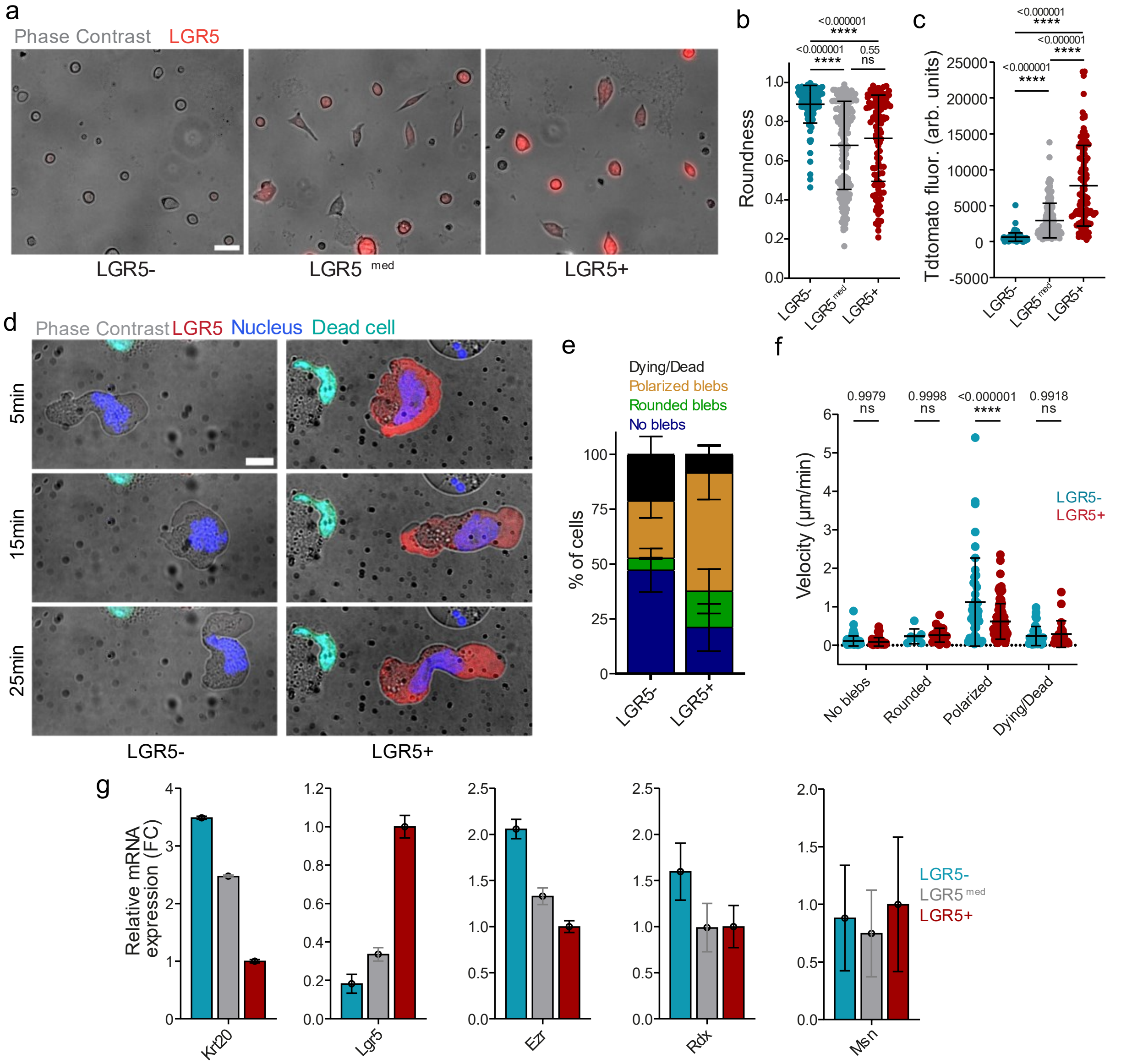

Supplementary Figure 3. PDO-p18 cells display mechanical phenotypes similar to PDO7 cells. **a.** LGR5-, LGR5^{med} and LGR5+ PDO-p18 cells on 3 kPa gel substrates. Representative images of two independent experiments. Scale bar, 20 µm. **b.** Cell roundness measured for sorted PDO-p18 single cells seeded on collagen-I coated gel substrates of 3 kPa in stiffness. **c.** TdTomato Fluorescence of LGR5-, LGR5^{med} and LGR5+ PDO-p18 cells. (**b, c**) Data are represented as the mean ± s.d. of n ≥113 cells/condition from two independent experiments. Statistical significance was determined using Shapiro-Wilk normality test, followed by a Kruskal-Wallis multiple-comparison test. **d.** Representative time lapse images of LGR5- no blebs, LGR5+ and LGR5- polarized blebs. Scale bar, 10 µm. Images are representative of four independent experiments. **e.** Percentage of dying/dead, polarized blebs, rounded blebs and no blebs in LGR5- and LGR5+ cells under 4.5 µm confinement on a non-adhesive surface. Data are represented as the mean ± s.d. of percentages from four independent experiments. Statistical significance was determined using two-way analysis of variance, followed by a Šidák multiple-comparison test. **f.** Migration velocity of tracked nuclei of LGR5- and LGR5+ cells, divided into categories according to the confinement response. Data are represented as the mean ± s.d. of n ≥ 183 cells/subgroup from four independent experiments. Statistical significance was determined using two-way

analysis of variance, followed by a Šidák multiple-comparison test. **g.** Relative mRNA expression levels of *Krt20*, *Lgr5*, and ERM proteins for sorted PDO-p18 cells. Data are represented as the mean ± s.d. of triplicates from one experiment. Source data are provided as a Source Data file.

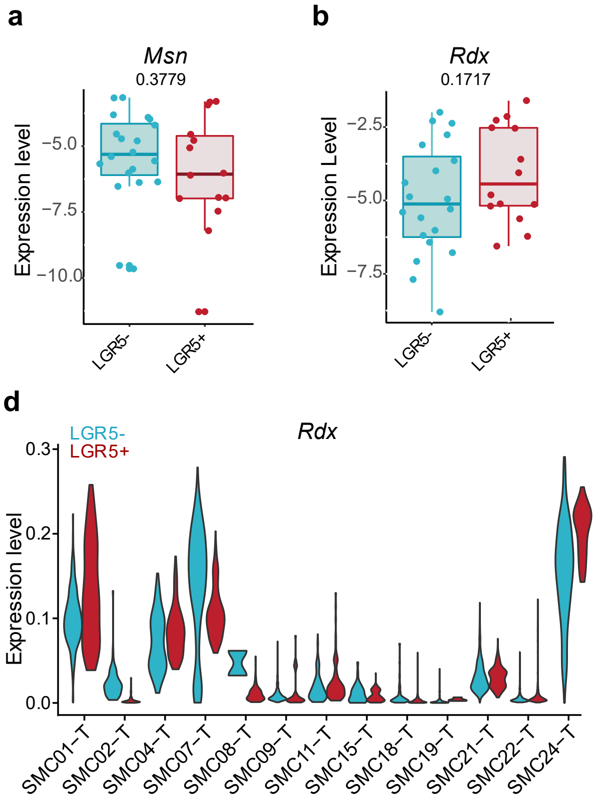

Supplementary Figure 4. **Expression of *Lgr5*, *Msn* and *Rdx* in CRC patients.** (**a, b**) Gene expression levels of *Rdx* and *Msn* in epithelial tumor cells from CRC patients in the SMC cohort summarized by patient through the average. Each dot corresponds to the average expression levels of one patient. summarized by patient through the average. The boxes center line represents the median. The box limits represent the first and third quartiles. Whiskers indicate maximum and minimum values. n = 15. A linear model was fitted to the data to assess significance. (**c-e**) Violin plots showing expression levels of *Lgr5* (**c**), *Rdx* (**d**) and *Msn* (**e**) in epithelial tumor cells from patients in the SMC cohorts. Patient ID is detailed on the horizontal axis.

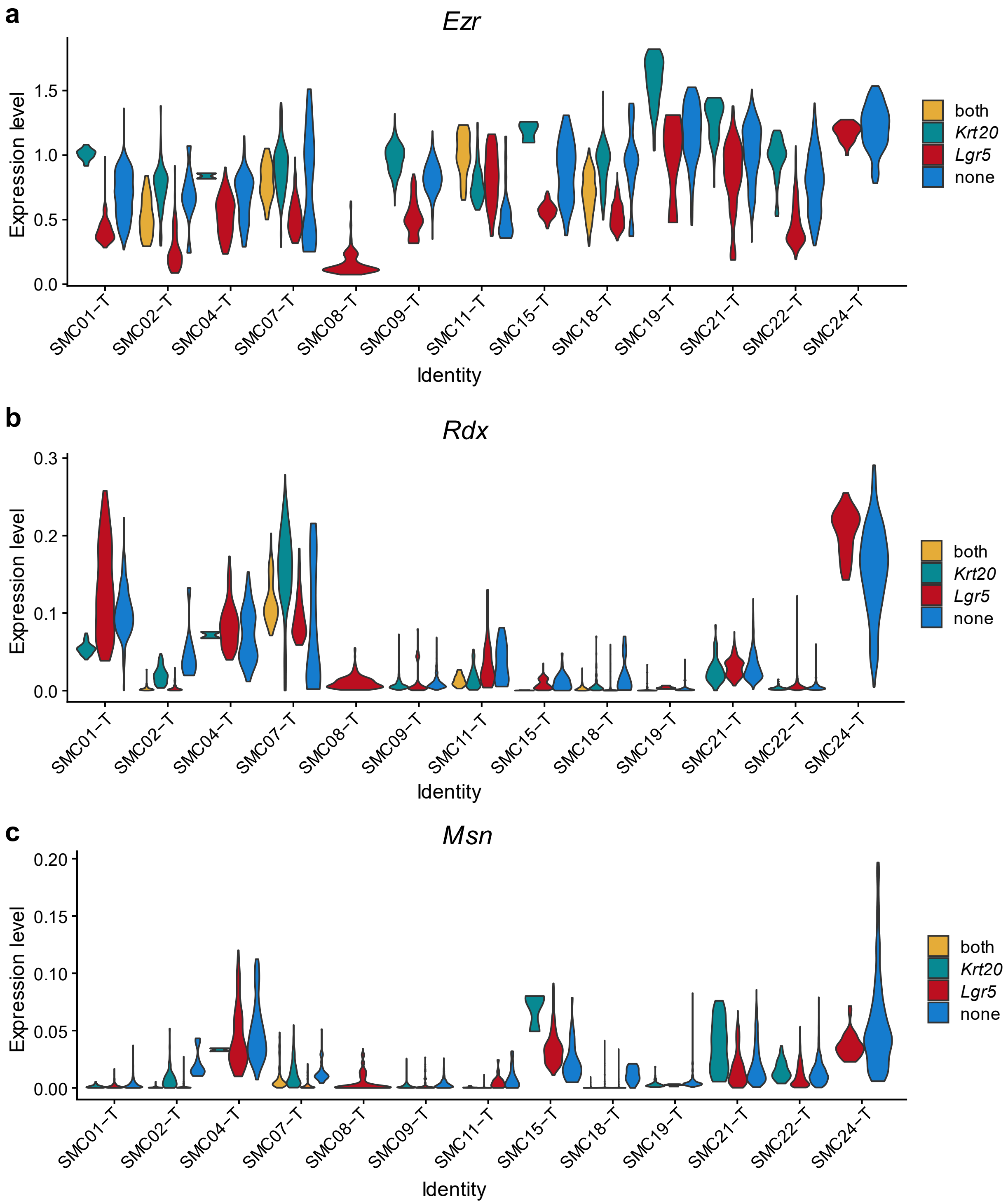

Supplementary Figure 5. Expression of ERM proteins. (**a-c**) Violin plots showing expression levels of *Ezr* (**a**), *Rdx* (**b**) and *Msn* (**c**) in epithelial tumor cells from patients in the SMC cohorts. Patient ID is provided in the horizontal axis. Cells were divided into four groups depending on whether they expressed *Lgr5* onl*y (red), Krt20* only (green), both (yellow) or none (blue).

Supplementary Data 1: Example of gating strategy

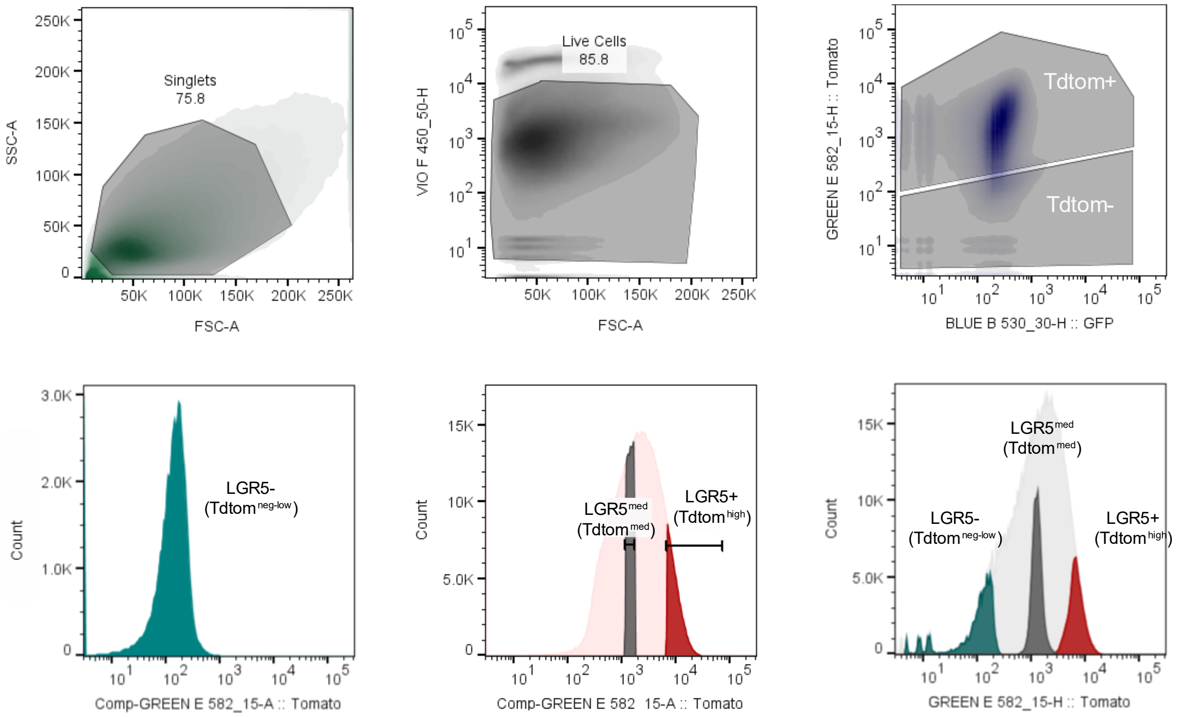

Supplementary Figure 6. Gating strategy for flow cytometry sorting of LGR5-, LGR5^{med} and LGR5+ cells.

Supplementary Tables

**Selected (Supp. Fig. 1d)**			
	**LGR5+ vs. LGR5-**	**LGR5+ vs. LGR5^{med}**	**LGR5^{med} vs LGR5-**
Differentiation (diffmix)	0.00029	0.22100	0.00037
GOBLET_Nusse_2018	0.00029	0.14538	0.00037
ENTEROCYTE_Nusse_2 018	0.00029	0.09419	0.00037
Mucosecreting_Hs	0.00029	0.00176	0.01232
TUFT_Nusse_2018	0.00029	0.14538	0.00037
coreHRC 100 (EpiHR)	0.00029	0.18411	0.00037
Fetal signature mustafa	0.00029	0.11702	0.00037
Lgr5.ISC.refined	0.00029	0.20591	0.02243
POLR1A.High	0.00029	0.14538	0.00037
Mex3aHI LGR5hi signature SI	0.00029	0.14538	0.03422
Proliferation	0.00029	0.14297	0.00037
Ki67_HvL	0.00029	0.10460	0.00037
**GOBP gene sets (Supp. Fig. 1e)**			
substrate adhesion-dependent cell spreading	0.00041	0.33544	0.00083
EMT	0.00041	0.23567	0.00083
epithelial cell migration	0.00041	0.31564	0.13335
cytoskeleton organization	0.00041	0.32075	0.05349
PM organization	0.00041	0.10397	0.11037
**GOCC gene sets (Fig. 5a)**			
anchored to membrane	0.00046	0.87661	0.00087
cell cortex	0.00040	0.36139	0.00087
actin filament bundle	0.02235	0.35013	0.05499
actin filament binding	0.00046	0.55087	0.11279
PDZ domain binding	0.00040	0.29591	0.00089
PIP_2 binding	0.00040	0.27463	0.20831

Supplementary Table 1. *p* values for Enrichment analysis Roast-GSA. Enrichment analysis was performed using ROAST-GSA methods that combines the statistical inference based on limma and rotations from ROAST and the re-standardized MaxMean statistic from GSA.

GeneName	Refseq	CloneID	Target Seq	OligoSeq	vectorID
VIL2	NM_003379.3	TRCN0000062460	CCCACGTCTGAGAATCAACAA	CCGGCCCACGTCTGAGAATCAACAACTCGAGTTGTTGATTCTCAGACGTGGGTTTTTG	pLKO.1
VIL2	NM_003379.3	TRCN0000062461	CGTGGGATGCTCAAAGATAAT	CCGGCGTGGGATGCTCAAAGATAATCTCGAGATTATCTTTGAGCATCCCACGTTTTTG	pLKO.1
RDX	NM_002906.3	TRCN0000062435	GCCAGAGATGAAACCAAGAAA	CCGGGCCAGAGATGAAACCAAGAAACTCGAGTTTCTTGGTTTCATCTCTGGCTTTTTG	pLKO.1
MSN	NM_002444.2	TRCN0000062411	GCATTGACGAATTTGAGTCTA	CCGGGCATTGACGAATTTGAGTCTACTCGAGTAGACTCAAATTCGTCAATGCTTTTTG	pLKO.1

Supplementary Table 2. shRNA sequences and vectors used to obtain silencing of ERM proteins.

Stiffness (kPa)	Acrylamide (BioRad) %	Bis-acrylamide (BioRad) %	Beads % solids	Ammonium persulphate (Sigma-Aldrich) %	Tetramethylethylene diamine (Sigma-Aldrich) %
0.5	4	0.03	0.03	0.5	0.05
1.5	5	0.04	0.03	0.5	0.05
3	6.16	0.044	0.03	0.5	0.05
5	7.46	0.044	0.03	0.5	0.05
11	7.5	0.1	0.03	0.5	0.05
30	12	0.15	0.03	0.5	0.05

Supplementary Table 3. Different recipes used for the PAA gel preparation.

Primer	Sequence
Lgr5 pair 1 forward	GCTTCCTGGAGGAGTTACGTC
Lgr5 pair 1 reverse	AACAGCTTGGGGGCACATAG
Krt20 pair 6 forward	CAGTGGTACGAAACCAACGC
Krt20 pair 6 reverse	TCCTCTCTCAGTCTCATACTTCAG
Ezr pair 1 forward	CTGCTCTGACTCCAGGTTGG
Ezr pair 1 reverse	GCCGATAGTCTTTACCACCTGAT
Rdx pair 7 forward	GATGAGTTTGAAGCAATGTGGGG
Rdx pair 7 reverse	TTAAGGCCCCAGAAAAACCCA
Msn pair 1 forward	CCATGCCCAAAACGATCAGTG
Msn pair 1 reverse	CAGCCAGGTGGAGAAACCTT

Supplementary Table 4. Primer sequences for RT-qPCR.
