# Supplementary material for: Membrane to cortex attachment determines different mechanical phenotypes in LGR5+ and LGR5- colorectal cancer cells
Source: Nat Commun. 2024 Apr 18;15:3363. doi: 10.1038/s41467-024-47227-2 (PMC11026456; doi:10.1038/s41467-024-47227-2)
Supplement: Supplementary file 2 — Description of Additional Supplementary Files [file 41467_2024_47227_MOESM2_ESM.pdf]

Supplementary Data

Supplementary Data 1: Enrichment analysis gene lists. Gene signatures lists used to characterize LGR5-, LGR5med and LGR5+ cell populations**.**

Supplementary Data 2: n values for main figures. Table detailing the number of cells per condition analyzed in each experiment**.**

Supplementary Movies

Supplementary Movie 1. CRC PDOs differentiate *in vitro*.
Confocal z-stack of a representative PDO cultured for 1 week in culture matrix gel stained for cytokeratin 20 (cyan). LGR5+ cells are labelled with Tdtomato (red).

Supplementary Movie 2. Response to confinement of LGR5+ and LGR5- cells.
Representative example of confined PDO single cells on a non-adhesive surface. LGR5+ cells are marked in red while LGR5- cells are unlabeled. All nuclei are labeled (blue). Images were acquired every 5 minutes for a total duration of 100 min.

Supplementary Movie 3. Response to confinement of LGR5+ and LGR5- cells.
Second representative example of confined PDO single cells on a non-adhesive surface. LGR5+ cells are marked in red while LGR5- cells are unlabeled. All nuclei are labelled (blue). Images were acquired every 5 minutes for a total duration of 100 min.

Supplementary Movie 4. Tracking of polarized LGR5- and LGR5+ cells.
Representative experiment. Left: LGR5+ cells are marked in red while LGR5- cells are unlabeled. All nuclei are labelled (blue). Right: cellular tracks overlaid on the phase contrast images. Images were acquired every 5 minutes for a total duration of 100 min. Note the fast LGR5- cell with a blue track.

Supplementary Movie 5. Tracking of polarized LGR5- and LGR5+ cells.
Second representative experiment. Left: LGR5+ cells are marked in red while LGR5- cells are unlabeled. All nuclei are labelled (blue). Right: cellular tracks overlaid on the phase contrast images. Images were acquired every 5 minutes for a total duration of 100 min.

Supplementary Movie 6. $LGR5^{high}$ cluster migrating on 2D soft substrate.
Left: Time lapse of $LGR5^{high}$ PDO cluster was acquired every hour for 15 hours. LGR5+ cells are marked in red while LGR5- cells are unlabeled. Right: Time lapse of cellular tractions corresponding to the cluster shown on the left panel. Colormap and traction vector scale are the same as in Fig. 3B.

Supplementary Movie 7. $LGR5^{low}$ cluster migrating on 2D soft substrate.
Left: Time lapse of $LGR5^{low}$ PDO cluster was acquired every hour for 15 hours. LGR5+ cells are marked in red while LGR5- cells are unlabeled. Right: Time lapse of cellular tractions corresponding to the cluster shown on the left panel. Colormap and traction vector scale are the same as in Fig. 3B.

Supplementary Movie 8. $LGR5^{low}$ cluster attaching to an endothelial monolayer.

Time lapse of LGR5^{low} PDO cluster seeded on top of a HUVEC monolayer. LGR5+ cells are marked in red and HUVEC cells are marked in green. Images were acquired 1 h after cluster seeding, every 40 minutes for 14 h.

Supplementary Movie 9. LGR5^{med} cluster attaching to an endothelial monolayer.
Time lapse of LGR5^{med} PDO cluster seeded on top of a HUVEC monolayer. LGR5+ cells are marked in red and HUVEC cells are marked in green. Images were acquired 1 h after cluster seeding, every 40 minutes for 14 h.

Supplementary Movie 10. LGR5^{high} cluster attaching to an endothelial monolayer.
Time lapse of LGR5^{high} PDO cluster seeded on top of a HUVEC monolayer. LGR5+ cells are marked in red and HUVEC cells are marked in green. Images were acquired 1 h after cluster seeding, every 40 minutes for 14 h.

Supplementary Movie 11. Response to confinement of LGR5+ cells expressing the synthetic iMC-linker and LGR5+ cells.
Confined PDO single cells on a non-adhesive surface. LGR5+ cells are marked in red. All nuclei are labelled (blue). Images were acquired every 5 minutes for a total duration of 70 min.
