# Supplementary material for: Membrane to cortex attachment determines different mechanical phenotypes in LGR5+ and LGR5- colorectal cancer cells
Source: Nat Commun. 2024 Apr 18;15:3363. doi: 10.1038/s41467-024-47227-2 (PMC11026456; doi:10.1038/s41467-024-47227-2)
Supplement: Supplementary file 16 — Reporting Summary [file 41467_2024_47227_MOESM16_ESM.pdf]

nature portfolio

Corresponding author(s): Xavier Trepat, Anna Labernadie, Eduard Batlle

Last updated by author(s): Jan 30, 2024

Reporting Summary

Nature Portfolio wishes to improve the reproducibility of the work that we publish. This form provides structure for consistency and transparency in reporting. For further information on Nature Portfolio policies, see our Editorial Policies and the Editorial Policy Checklist.

Statistics

For all statistical analyses, confirm that the following items are present in the figure legend, table legend, main text, or Methods section.

n/a	Confirmed	
☐	☒	The exact sample size (*n*) for each experimental group/condition, given as a discrete number and unit of measurement
☐	☒	A statement on whether measurements were taken from distinct samples or whether the same sample was measured repeatedly
☐	☒	The statistical test(s) used AND whether they are one- or two-sided<br>*Only common tests should be described solely by name; describe more complex techniques in the Methods section.*
☒	☐	A description of all covariates tested
☐	☒	A description of any assumptions or corrections, such as tests of normality and adjustment for multiple comparisons
☐	☒	A full description of the statistical parameters including central tendency (e.g. means) or other basic estimates (e.g. regression coefficient) AND variation (e.g. standard deviation) or associated estimates of uncertainty (e.g. confidence intervals)
☐	☒	For null hypothesis testing, the test statistic (e.g. *F*, *t*, *r*) with confidence intervals, effect sizes, degrees of freedom and *P* value noted<br>*Give P values as exact values whenever suitable.*
☒	☐	For Bayesian analysis, information on the choice of priors and Markov chain Monte Carlo settings
☒	☐	For hierarchical and complex designs, identification of the appropriate level for tests and full reporting of outcomes
☒	☐	Estimates of effect sizes (e.g. Cohen's *d*, Pearson's *r*), indicating how they were calculated

Our web collection on statistics for biologists contains articles on many of the points above.

Software and code

Policy information about availability of computer code

Data collection	Image acquisition: Nikon NIS-Elements and MicroManager (version 1.4.22). Flow cytometry and cell sorting: BD FACS DIVA V8.0.1. Real time PCR: Step one software v2.3.
Data analysis	MATLAB. version 9.6.0.1072779 (R2019a), MathWorks Inc., Natick, Massachusetts, 2019. Costum made codes can be made available upon request to the corresponding authors on reasonable request. GraphPad Prism 9 was used for graph generation and for statistical analysis. Image/ Fiji was used for some image analysis and/or image processing. FlowJo (v10.7) and BD FACS DIVA were used for flow cytometry data analysis.

For manuscripts utilizing custom algorithms or software that are central to the research but not yet described in published literature, software must be made available to editors and reviewers. We strongly encourage code deposition in a community repository (e.g. GitHub). See the Nature Portfolio guidelines for submitting code & software for further information.

Data

Policy information about availability of data

All manuscripts must include a data availability statement. This statement should provide the following information, where applicable:

- Accession codes, unique identifiers, or web links for publicly available datasets
- A description of any restrictions on data availability
- For clinical datasets or third party data, please ensure that the statement adheres to our policy

The RNA-seq data generated in this study are available at the Gene Expression Omnibus (GEO) with the accession number: GSE247359. Previously published single

cell RNA sequencing data of CRC patient samples were reanalyzed and are available at GEO under accession codes GSE13246575. Count matrices for single-cell RNA-seq experiments were deposited at ArrayExpress under accession number E-MTAB-8107 and E-MTAB-9934. Data from healthy human samples can be found in the gut atlas human dataset (https://www.gutcellatlas.org), with accession code E-MTAB-8901 (https://www.ebi.ac.uk/biostudies/arrayexpress/studies/E-MTAB-8901).

Research involving human participants, their data, or biological material

Policy information about studies with human participants or human data. See also policy information about sex, gender (identity/presentation), and sexual orientation and race, ethnicity and racism.

Reporting on sex and gender	*Use the terms sex (biological attribute) and gender (shaped by social and cultural circumstances) carefully in order to avoid confusing both terms. Indicate if findings apply to only one sex or gender; describe whether sex and gender were considered in study design; whether sex and/or gender was determined based on self-reporting or assigned and methods used. Provide in the source data disaggregated sex and gender data, where this information has been collected, and if consent has been obtained for sharing of individual-level data; provide overall numbers in this Reporting Summary. Please state if this information has not been collected. Report sex- and gender-based analyses where performed, justify reasons for lack of sex- and gender-based analysis.*
Reporting on race, ethnicity, or other socially relevant groupings	*Please specify the socially constructed or socially relevant categorization variable(s) used in your manuscript and explain why they were used. Please note that such variables should not be used as proxies for other socially constructed/relevant variables (for example, race or ethnicity should not be used as a proxy for socioeconomic status). Provide clear definitions of the relevant terms used, how they were provided (by the participants/respondents, the researchers, or third parties), and the method(s) used to classify people into the different categories (e.g. self-report, census or administrative data, social media data, etc.) Please provide details about how you controlled for confounding variables in your analyses.*
Population characteristics	The PDO model used in most of the in vitro experiments has been previously described and referred to as PDO7 in Cortina et al. 2017 and Lombardo et al. 2011. PDO-p18 is described in van de Wetering et al. 2015. The two models were obtained from from CRC patients undergoing CR resection, in accordance with the ethical standards of the institutional committee.
Recruitment	*Describe how participants were recruited. Outline any potential self-selection bias or other biases that may be present and how these are likely to impact results.*
Ethics oversight	*Identify the organization(s) that approved the study protocol.*

Note that full information on the approval of the study protocol must also be provided in the manuscript.

Field-specific reporting

Please select the one below that is the best fit for your research. If you are not sure, read the appropriate sections before making your selection.

☒ Life sciences ☐ Behavioural & social sciences ☐ Ecological, evolutionary & environmental sciences

For a reference copy of the document with all sections, see nature.com/documents/nr-reporting-summary-flat.pdf

Life sciences study design

All studies must disclose on these points even when the disclosure is negative.

Sample size	No statistical test was used to determine sample size upfront. Instead sample size was determined empirically according to previous knowledge of the variation similar experimental setups. For the majority of the experiments a minimum of n=2 independent experiments were performed, with multiple measurements per experiment, which is sufficient to detect meaningful differences based on historical record with similar experiments.
Data exclusions	No data were excluded from this study.
Replication	The majority of experiments were replicated at least twice. Experiments that were replicated twice were:<br>Immunostaining of PDOs for CK20 expression (Fig. 1a)<br>Actin staining of PDO clusters on 3kPa gels for the calculation of contact angle (Fig. 3h)<br>Western for Total ERMs and phosphoERMs (Fig. 5c)<br>Morphology assessment of single PDO cells (LGR5+, LGR5-, iMC-linker expressing LGR5+ cells, ERM silenced LGR5- cells)- (Fig. 5f, g).<br>Migration velocity of LGR5high, LGR5low, iMC-linker expressing LGR5high clusters and LGR5low clusters with ERM silencing. (Fig. 5j).<br><br>The Bulk RNAseq experiment was performed once using two replicates for each sample.<br>Replication of the data was at all times successful. Experiments were performed at different days and using different batches of PDOs, obtaining the same results.
Randomization	In experiments involving comparison between groups, no particular method was used to randomize the experiments. Experimental groups for comparisons between LGR5+ and LG5med and LGR5- were allocated based on objective measurements of cell fluorescence, which prevents experimental biases.

Blinding | Blinding was not possible as the same researcher executed and analyzed the experiments.

Reporting for specific materials, systems and methods

We require information from authors about some types of materials, experimental systems and methods used in many studies. Here, indicate whether each material, system or method listed is relevant to your study. If you are not sure if a list item applies to your research, read the appropriate section before selecting a response.

Materials & experimental systems

n/a	Involved in the study
☐	☒ Antibodies
☐	☒ Eukaryotic cell lines
☒	☐ Palaeontology and archaeology
☒	☐ Animals and other organisms
☒	☐ Clinical data
☒	☐ Dual use research of concern
☒	☐ Plants

Methods

n/a	Involved in the study
☒	☐ ChIP-seq
☐	☒ Flow cytometry
☒	☐ MRI-based neuroimaging

Antibodies

Antibodies used

The following is a list of the primary antibodies used and their respective dilutions were: mouse anti-YAP, 1:200 (Santa Cruz, cat. no. sc-271134); mouse anti-CK20, 1:100 (Dako, cat. no. M7019); rabbit anti-VE-cadherin, 1:2,000 (Invitrogen, cat. no. PA5-19612), rabbit anti-pMLC P-Myosin Light Chain 2 (Thr18/Ser19), 1:200 (Cell Signaling cat. no. 3674), rabbit anti-. Ezrin/Radixin/Moesin, 1:100 (Cell Signaling cat. no. 3142), rabbit anti-phospho Ezrin (Thr567)/ Radixin (Thr564)/ Moesin (Thr558), 1:100 (Cell Signaling cat. no. 3141).

The secondary antibodies used were: goat anti-mouse Alexa Fluor 488 (Thermo Fisher Scientific, cat. no. A-11029); donkey anti-rabbit Alexa Fluor 488 (Thermo Fisher Scientific, cat. no. A-21206), goat anti-rabbit Alexa Fluor 555 (Thermo Fisher Scientific, cat. no. A-21429) and goat anti-mouse Alexa Fluor 405 (Abcam, cat. no. ab175660). All of the secondary antibodies were used at a dilution of 1:400. To label F-actin, phalloidin Atto 488 (Sigma-Aldrich cat. no. 49409) was used at 1:500 and phalloidin Alexa Fluor-647 (Thermo Fisher Scientific, cat. no. A22287) at 1:400. Hoechst (Thermo Fisher Scientific, cat. no. 33342) was used to label nuclei.

Validation

All the antibodies were used for immunofluorescence. They were all validated by their respective manufacturers and by previous studies. Previous validations and citations can be found by using the following RRID numbers or Labome reference:
mouse anti-YAP - AB_10612397 https://www.antibodyregistry.org/AB_10612397
mouse anti-CK20- M7019- Ks20.8. https://www.labome.com/product/Dako/M7019.html
rabbit anti-VE-cadherin - AB_10979589 https://www.antibodyregistry.org/AB_10979589
rabbit anti-pMLC P-Myosin Light Chain 2 (Thr18/Ser19) - AB_2147464 https://www.antibodyregistry.org/AB_2147464
rabbit anti-. Ezrin/Radixin/Moesin - AB_2100313 https://www.antibodyregistry.org/AB_2100313
rabbit anti-phospho Ezrin (Thr567)/ Radixin (Thr564)/ Moesin (Thr558) - AB_330232 https://www.antibodyregistry.org/AB_330232

goat anti-mouse Alexa Fluor 488 - AB_2534088 https://www.antibodyregistry.org/AB_2534088
donkey anti-rabbit Alexa Fluor 488 - AB_2535792 https://www.antibodyregistry.org/AB_2535792
goat anti-rabbit Alexa Fluor 555 - AB_2535850 https://www.antibodyregistry.org/AB_2535850
goat anti-mouse Alexa Fluor 405 - AB_2885184 https://www.antibodyregistry.org/AB_2885184

Eukaryotic cell lines

Policy information about cell lines and Sex and Gender in Research

Cell line source(s)	293 [HEK-293] is a cell line that was isolated from the kidney of a human embryo.
Authentication	none of the cell lines were authenticated
Mycoplasma contamination	All the cell lines used tested negative for Mycoplasma.
Commonly misidentified lines (See ICLAC register)	*Name any commonly misidentified cell lines used in the study and provide a rationale for their use.*

Plants

Seed stocks	*Report on the source of all seed stocks or other plant material used. If applicable, state the seed stock centre and catalogue number. If plant specimens were collected from the field, describe the collection location, date and sampling procedures.*
Novel plant genotypes	*Describe the methods by which all novel plant genotypes were produced. This includes those generated by transgenic approaches, gene editing, chemical/radiation-based mutagenesis and hybridization. For transgenic lines, describe the transformation method, the number of independent lines analyzed and the generation upon which experiments were performed. For gene-edited lines, describe the editor used, the endogenous sequence targeted for editing, the targeting guide RNA sequence (if applicable) and how the editor was applied.*
Authentication	*Describe any authentication procedures for each seed stock used or novel genotype generated. Describe any experiments used to assess the effect of a mutation and, where applicable, how potential secondary effects (e.g. second site T-DNA insertions, mosiacism, off-target gene editing) were examined.*

Flow Cytometry

Plots

Confirm that:

☒ The axis labels state the marker and fluorochrome used (e.g. CD4-FITC).

☒ The axis scales are clearly visible. Include numbers along axes only for bottom left plot of group (a 'group' is an analysis of identical markers).

☒ All plots are contour plots with outliers or pseudocolor plots.

☒ A numerical value for number of cells or percentage (with statistics) is provided.

Methodology

Sample preparation	The organoid-containing drops were enzymatically dissociated by TrypLE (Gibco) for 15 minutes at 37° C and reduced to single cell suspension by pipetting. TrypLE was then diluted with washing medium (Advanced DMEM/F12, 10 mM HEPES (Sigma-Aldrich), 1% GlutaMax) and centrifuged at 100g at RT for 3.5 min. PDOs were dissociated as described above and resuspended with cold tumor organoid medium at a concentration of 1x106 cells ml-1. Single cells suspension was stained with Dapi for 10 minutes, then analyzed and sorted.
Instrument	FACSAriaFusion flow cytometer (Beckton Dickinson).
Software	BD FACS diva siftware.
Cell population abundance	Purity was not assessed.
Gating strategy	Organoid populations were gated as follow: Cells/ Single Cells/ Living Cells/ Tdtomato+ cells or Tdtomatomed cells or Unlabelled cells. We defined the Tdtomto+ cells (LGR5+) as the 10% brightest cells.

☒ Tick this box to confirm that a figure exemplifying the gating strategy is provided in the Supplementary Information.
